# Urban environment as an independent predictor of insulin resistance in a South Asian population

**DOI:** 10.1186/s12942-019-0169-9

**Published:** 2019-02-12

**Authors:** Mohan Thanikachalam, Christina H. Fuller, Kevin J. Lane, Jahnavi Sunderarajan, Vijayakumar Harivanzan, Doug Brugge, Sadagopan Thanikachalam

**Affiliations:** 1 Department of Public Health and Community Medicine, Tufts University School of Medicine, 136 Harrison Avenue, Boston, MA 02111 USA; 2 Department of Population Health Sciences, Georgia State University School of Public Health, Atlanta, GA USA; 3 Department of Environmental Health, Boston University School of Public Health, Boston, MA USA; 4 Department of Biostatistics, Agada Hospital, Chennai, India; 5 PURSE-HIS Study Center, Sri Ramachandra University, Chennai, India; 6 Department of Civil and Environmental Engineering, Tufts University School of Engineering, Medford, MA USA; 7 Jonathan M. Tisch College of Civic Life, Tufts University, Medford, MA USA

**Keywords:** Urban, Insulin resistance, Diabetes, India, HOMA-IR

## Abstract

**Background:**

Developing countries, such as India, are experiencing rapid urbanization, which may have a major impact on the environment: including worsening air and water quality, noise and the problems of waste disposal. We used health data from an ongoing cohort study based in southern India to examine the relationship between the urban environment and homeostasis model assessment of insulin resistance (HOMA-IR).

**Methods:**

We utilized three metrics of urbanization: distance from urban center; population density in the India Census; and satellite-based land cover. Restricted to participants without diabetes (N = 6350); we built logistic regression models adjusted for traditional risk factors to test the association between urban environment and HOMA-IR.

**Results:**

In adjusted models, residing within 0–20 km of the urban center was associated with an odds ratio for HOMA-IR of 1.79 (95% CI 1.39, 2.29) for females and 2.30 (95% CI 1.64, 3.22) for males compared to residing in the furthest 61–80 km distance group. Similar statistically significant results were identified using the other metrics.

**Conclusions:**

We identified associations between urban environment and HOMA-IR in a cohort of adults. These associations were robust using various metrics of urbanization and adjustment for individual predictors. Our results are of public health concern due to the global movement of large numbers of people from rural to urban areas and the already large burden of diabetes.

**Electronic supplementary material:**

The online version of this article (10.1186/s12942-019-0169-9) contains supplementary material, which is available to authorized users.

## Background

Currently, 54% of the world’s population lives in urban areas, a proportion that is expected to increase to 66% by 2050 [1]. Most of the expected urban growth will take place in developing countries in Asia and Africa. Next to China, the world’s second largest urban population resides in India with approximately 410 million people and this number is projected to double by 2050 [1].

India had over 69.2 million people living with diabetes in 2015, and this number is expected to grow to 123.5 million by 2040 [2, 3]. In India, urban compared to rural populations have significantly higher diabetes prevalence [4, 5]. Studies have shown that urbanization in India is associated with increased consumption of energy-rich foods and a decrease in energy expenditure (through less physical activity) leading to obesity and increased risk of developing type 2 diabetes mellitus (diabetes) and other cardiometabolic conditions [5–8]. Rapid urbanization in India also often coincides with increased environmental pollution with potential harmful effects to health due to undesirable changes in the physical, chemical or biological characteristics of air, water or land [9]. Emerging epidemiologic data suggests that environmental pollutants could be a risk factor for diabetes [10, 11].

In a study in Chennai the overall diabetes prevalence increased from 11.6% in 1995 to 13.9% in 2000 [15]. Chennai, located in the rapidly urbanizing southern state of Tamil Nadu, is the fourth largest metropolitan city in India. Subsequent studies of adults over 20 years old in Chennai showed that the prevalence of diabetes increased from 14.3% in 2003–2004 to 18.6% in 2006 [3, 4]. In a more recent study (2010–2011) the age standardized prevalence of diabetes in Chennai was 22.8% (95% CI 21.5–24.1%) [12]. These results indicate a rapid increase in prevalence of diabetes in Chennai city in recent decades [3, 4, 12, 13]. In a comparative study, residents in Chennai had lower BMI and waist circumference (WC) measurements than Asian Indians living in the U.S., but still had a higher prevalence of diabetes even at normal levels of BMI [14]. Adjustment for age, sex, WC, and systolic blood pressure did not fully explain differences in the odds of diabetes between the two groups suggesting that factors besides age and central adiposity play a role in diabetes development.

There is minimal data on urban environmental degradation and risk for diabetes in developing countries such as India [9]. Insulin resistance, which is a reduction in the cellular response to endogenous insulin, is a powerful predictor of future development for diabetes [15]. Studies have shown links between insulin resistance and various chemicals, such as phthalates and bisphenol A (BPA), found in polluted environments [16–18]. Animal and recent epidemiological studies have reported that air pollutants, such as, nitrogen dioxide (NO_2_) and PM_2.5_ may affect insulin sensitivity [19–21]. Given the high levels of environmental pollutants in India, it is plausible that some of these pollutants could be factors within the urban environment contributing to increased diabetes risk [5].

In the current study, while controlling for traditional risk factors, we examine the cross sectional association between insulin resistance and measures of urban environment defined using the following metrics: (1) distance from urban center; (2) population density in the India Census; and (3) satellite-derived land cover type. We used health data from the Population Study of Urban, Rural, and Semi-urban Regions for the Detection of Endovascular Disease and Prevalence of Risk Factors and Holistic Intervention Study (PURSE-HIS) in a population recruited from Chennai and surrounding areas [22].

## Methods

The PURSE-HIS was designed and implemented to understand the prevalence and progression of subclinical and overt cardiovascular disease (CVD) and its risk factors in urban, semi-urban, and rural communities in southern India. Detailed methodology has been published elsewhere [22]. Briefly, Chennai served as the primary location from which the urban study population was recruited. The semi-urban and rural areas were near Chennai in the Thiruvallur and Kanchipuram districts, respectively. A total of 8080 participants over 20 years of age were recruited between 2009 and 2011 from urban (N = 2221), semi-urban (N = 2821) and rural (N = 3038) areas. A two stage cluster sampling method was used to ensure adequate spatial variability amongst administrative divisions. After excluding participants with a previous history of diabetes or newly diagnosed diabetes (a fasting blood glucose ≥ 126 mg/dL or a 2-h oral glucose tolerance test (OGTT) ≥ 200 mg/dL) our sample size was 6350; which included 3670 females and 2680 males.

### Questionnaire and clinical data collection

An interviewer-administered questionnaire was used to collect data on demographics, CVD and its risk factors [22]. Physical activity was measured by a physiotherapist using the Global Physical Activity Questionnaire [23] and a score was calculated. A clinical psychologist assessed the level of stress and anxiety levels using the Presumptive Stressful Life Event Scale [24]. A socioeconomic (SE) score was computed based on a revision of the Kuppussamy classification scale [25, 26]. Kuppuswamy’s SE score was originally proposed in 1976 and was built for the Indian population combining values for education, occupation, education and income to create a robust estimate of standard of living. Participants are categorized into lower, middle and upper classes. Energy (food) intake was assessed from a 24-h recall of meals and a food frequency questionnaire [27]. Body mass index (BMI) was calculated by dividing the participant’s measured weight in kilograms by the square of height in meters. Fasting blood specimens were collected and assayed for fasting blood sugar (FBS) and fasting insulin levels [22]. Homeostasis model assessment of insulin resistance (HOMA-IR) was calculated as fasting plasma insulin (mU/L) × FBS (mmol/L)/22.5. Since a diagnostic test for insulin resistance does not exist, insulin resistance was defined as a HOMA-IR level above the 75th percentile, as previously defined in multiple cohort and epidemiological studies [28].

### Geo-location and creation of urbanization metrics

**Fig. 1:**
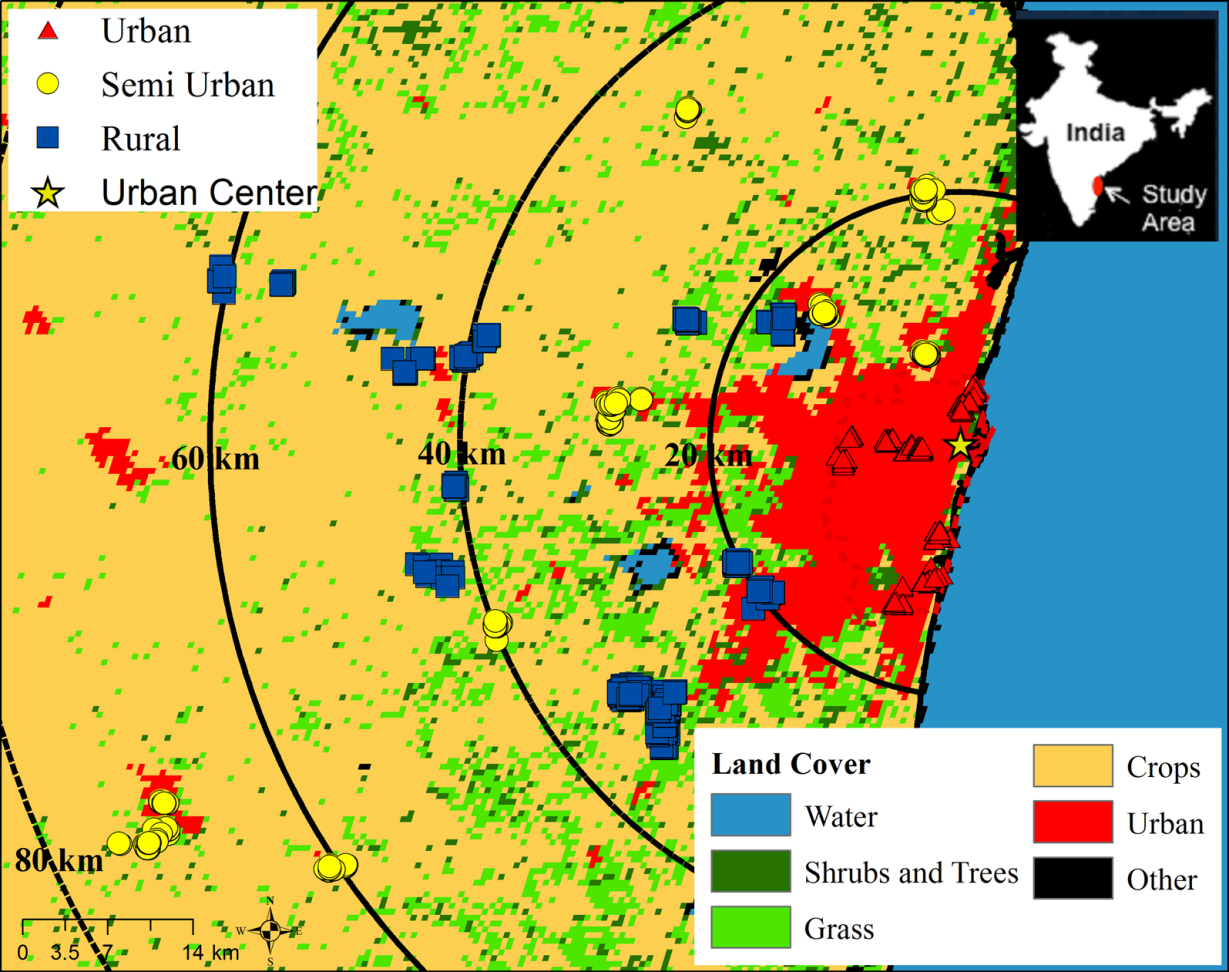
Land cover and distance from urban center with PURSE-HIS participants

We defined urbanization using three different metrics: distance from urban center (Chennai), land cover type, and census community designation. Residential addresses of study participants were geolocated to the nearest road or intersection through manual assignment by a single researcher using Google Earth© over the study area that spanned a geographic region of approximately 80 km by 80 km (Fig. 1). For quality control purposes, the Google Earth© location identification process was repeated by a second researcher with 100 randomly selected participants to examine potential positional error. We found minimal error in the geolocation of participants. The average difference in distance for the randomly selected participants was 0.18 km with a minimum of 0.01 km and a maximum of 0.42 km.

For the first metric, the urban center of the study region was defined as the flag post on the ramparts of the Fort Saint George historic landmark in Chennai, in accordance with historical and local custom. Residential location KML files were imported into ArcGIS v10.1 (ESRI, Redlands, CA) to calculate the distance, in kilometers, and compass angle from the urban center for each participant using the Near Tool.

The second metric utilized land cover data (MCD12Q1, NASA) obtained through the online Data Pool at the NASA Land Processes Distributed Active Archive Center. The values were derived from Terra and Aqua-MODIS land cover data products which provided yearly averages [29]. The data presented are from the year 2010 and have 500 m × 500 m resolution. We based our groups on the 17 land cover classifications of the International Geosphere Biosphere Program Plant Functional Scheme, which together have 72–77% classification accuracy [30]. We mapped the classifications as five distinct groups, which included urban, trees/shrubs, grass, crops, and other (Fig. 1). (The figure also includes water for illustration; however, no participants resided in these grids.) We then aggregated all the non-urban land classifications into a single group, which we designated as rural and the remaining groups as urban.

The third metric, census community designation, was based on data from the 2011 India census. Participants residing in urban areas were those living in a municipality with a total population of at least 5000 and a population density of 400 persons/km^2^ or more. Those residing in municipalities with smaller populations or densities were designated as non-urban [31].

### Statistical analyses

We first evaluated descriptive statistics for population characteristics according to urban designation and separated by gender. Analysis of variance was used to check for significant differences on mean scores in both genders between rural and urban residents. Analysis of variance was also used to test for significant differences in HOMA-IR scores according to categories of age (≤ 39 or ≥ 40), body mass index (non-obese [BMI ≤ 24.9] or obese [BMI ≥ 25]), physical activity (low, moderate or high), SE score (lower, middle or upper) and smoking (smoker or non-smoker). We ran logistic regression models to evaluate the association between each urbanization metric and the odds of having a HOMA-IR level in the fourth quartile of the distribution.

Logistic regression models were adjusted for age, BMI, physical activity, energy intake, SE score and smoking in separate models for males and females. However, no adjustment for smoking was made in the models for females due to the very low prevalence of smoking. We evaluated effect modification, in models with distance to urban center as the exposure, by stratifying on categories of age, smoking status, BMI and physical activity. Potential modifiers were removed as covariates in the model as appropriate when evaluating modification. The standardized coefficients and 95% confidence intervals were multiplied by the interquartile range (IQR) (i.e. 32.6 km). We also conducted a sensitivity analysis that substituted WC for BMI in adjusted regression models.

## Results

Population characteristics are given in Table 1. The mean age for females was 40 years and for males was 45 years. In both females and males, when compared to the rural population, the urban population had a significantly higher energy intake, SE score, stress score, insulin level and HOMA-IR. The urban population also had a higher BMI and was less physically active. The prevalence of smoking was higher in rural males compared to urban males and despite an overall low prevalence, smoking was higher among urban females compared to rural females.

**Table 1:** Population characteristics by sex and urban designation

Characteristics	Females		Males	
	Rural (N = 1467)	Urban (N = 2203)	Rural (N = 1054)	Urban (N = 1626)
Age (years)^b^	40.1 (9.2)	40.3 ± 9.5	45.4 (10.7)	44.1 (10.4)
FBS (mg/dL)^a^	94.5 (9.6)	92.4 (8.6)	92.6 (9.6)	92.2 (9.7)
Insulin (mIU/L)^a,b^	7.87 (5.24)	8.93 (7.58)	6.45 (4.66)	8.02 (6.07)
HOMA-IR (mg/dL)^a,b^	1.87 (1.29)	2.06 (1.79)	1.49 (1.09)	1.85 (1.53)
Energy intake (kcal)^a,b^	2338.4 (685.6)	2446.9 (704.2)	2952.0 (807.7)	3210.4 (928.1)
BMI ≤ 24.9^a,b^	852 (58.1%)	938 (42.6%)	790 (75%)	1011 (62.2%)
BMI ≥ 25^a,b^	615 (41.9%)	1265 (57.4%)	264 (25%)	615 (37.8%)
Socioeconomic score^a,b^	11.9 (4.2)	12.7 (4.2)	13.2 (4.5)	14.2 (4.9)
Stress score^a,b^	4.1 (3.4)	4.9 (3.0)	4.5 (3.2)	5.1 (2.7)
Physical activity				
Low^a,b^	207 (14.1%)	368 (16.7%)	242 (23%)	481 (29.6%)
Moderate	1194 (81.4%)	1804 (81.9%)	680 (64.5%)	1044 (64.2%)
High^a,b^	64 (4.4%)	31 (1.4%)	132 (12.5%)	100 (6.2%)
Smoking				
Smokers^a,b^	9 (0.6%)	70 (3.2%)	422 (40%)	444 (27.3%)
Non-smokers^a,b^	1458 (99.4%)	2133 (96.8%)	632 (60%)	1182 (72.7%)

^a^Sig. difference between rural and urban females

^b^Sig. difference between rural and urban males

Table 2 shows HOMA-IR levels stratified by demographic and urbanization variables. The overall mean HOMA-IR levels were 1.98 ± 1.61 for females and 1.71 ± 1.39 for males. HOMA-IR levels in both females and males were significantly higher in sub-populations with low and moderate physical activity compared to high physical activity. HOMA-IR levels were significantly higher among non-smoking males compared to smoking males and also higher for participants who were obese. There were no statistically significant differences by age, however, mean HOMA-IR was slightly higher for older females and younger males. HOMA-IR levels were higher for residents of urban areas compared to non-urban. Mean HOMA-IR levels were 2.69 ± 2.44 for females and 2.39 ± 2.29 for males living within 0–20 km from city center. This was statistically significantly higher than their counterparts living at a greater distance. Urban designation compared to rural according to land area was statistically significantly higher among females and males. Similarly, census derived urban residence was statistically significantly higher for females and males compared to rural populations.

**Table 2:** Mean HOMA-IR (mg/dL) stratified by demographic and urbanization variables

Characteristic	Females		Males	
	Mean (SD)	Min–max	Mean(SD)	Min–max
All participants	1.98 (1.61)	0.15–45.88	1.71 (1.39)	0.02–32.00
Age (years)				
≤ 39	1.96 (1.48)	0.15–35.13	1.75 (1.20)	0.02–7.93
≥ 40	2.01 (1.73)	0.18–35.75	1.69 (1.47)	0.06–32.00
BMI(kg/m^2^)^a^				
Non-obese ≤ 24.9	1.52 (1.32)	0.15–35.13	1.31 (1.01)	0.02–16.28
Obese ≥ 25	2.42 (1.75)	0.43–35.75	2.53 (1.67)	0.19–32.00
Physical activity^b,c^				
Low	2.11 (1.81)	0.18–35.75	2.06 (1.43)	0.12–12.44
Moderate	1.97 (1.59)	0.15–35.13	1.63 (1.39)	0.02–32.00
High	1.54 (0.86)	0.41–5.57	1.25 (0.97)	0.11–7.03
Smoking status^d^				
Smoker	1.96 (0.99)	0.59–4.86	1.54 (1.63)	0.02–32.00
Non-smoker	1.98 (1.63)	0.15–35.75	1.79 (1.24)	0.09–16.28
Distance Urban Center (km)^e,f^				
0–20 (0)	2.69 (2.44)	0.15–35.75	2.39 (2.29)	0.02–32.00
20–40 (1)	2.35 (2.15)	0.19–45.88	2.13 (2.11)	0.19–29.50
40–60 (2)	2.04 (1.48)	0.18–11.47	1.93 (1.80)	0.11–18.06
60–80 (3)	2.12 (1.93)	0.23–31.56	1.93 (1.82)	0.17–22.34
Land cover category^g^				
Urban	2.68 (2.48)	0.20–35.13	2.44 (2.27)	0.06–32.00
Non-Urban	2.28 (2.01)	0.15–45.88	2.04 (2.01)	0.02–29.5
Census Designation^h^				
Urban urban	2.53 (2.53)	0.19–35.75	2.35 (2.22)	0.02–32.00
Rural	2.20 (2.05)	0.15–45.88	1.87 (1.85)	0.11–29.50

^a^Statistically significant difference (p < 0.01) between non-obese and obese among females and males

^b^Statistically significant difference (p < 0.05) between low & high and moderate & high physical activity among females

^c^Statistically significant difference (p < 0.05) between low & moderate, low & high, and moderate & high physical activity among males

^d^Statistically significant difference (p < 0.05) between smokers and non-smokers among males

^e^Statistically significant difference between 0&1, 0&2, 0&3 (p < 0.001) and 1&2 in distance categories among females

^f^Statistically significant difference between 0&1 (p < 0.05), 0&2, 0&3 (p < 0.001) in distance categories among males

^g^Statistically significant difference between urban and non-urban (p < 0.001) among males and females

^h^Statistically significant difference between rural and urban (p < 0.001) among males and females

Results of unadjusted and adjusted logistic regression models of HOMA-IR are given in Table 3. Adjustment for potential confounders resulted in an attenuation of the effect in most cases. The OR for high HOMA-IR appeared to increase as distance to urban center decreased. In adjusted models, using distance from urban center, residing within 0–20 km was associated with an OR for high HOMA-IR of 1.79 (95% CI 1.39, 2.29) for females and 2.3 (95% CI 1.64, 3.22) for males compared to residing in the furthest 61–80 km group. Increased ORs ratios were also found for females living in the 20–40 km distance category (1.72 [95% CI 1.39, 2.29]) and for males living at distances of 20–40 km (1.59 [95% CI 1.11, 2.29]) and 41–60 km (1.77 [95% CI 1.19, 2.64]). Based on land cover, urban females (1.31 [95% CI 1.10, 1.50]) had significantly higher OR for HOMA-IR, while the effect for urban males was higher, but not statistically significant (1.20 [95% CI 0.98, 1.60]). Based on census community designation, the adjusted models showed that residing in an urban area resulted in a significantly higher OR in males (1.35 [95% CI 1.07, 1.72]), but not females (1.04 [95% CI 0.88, 1.24]).

**Table 3:** Odds ratio (OR) for insulin resistance (75th percentile HOMA-IR is 2.35)

Urbanization metric	Females		Males	
	OR unadjusted (95% CI)	OR adjusted (95% CI)	OR unadjusted (95% CI)	OR adjusted (95% CI)
Distance from urban center (km)				
< 20	*1.95 (1.55, 2.45)*	*1.79 (1.39, 2.29)*	*2.17 (1.63, 2.90)*	*2.30 (1.64, 3.22)*
20–40	*1.79 (1.42, 2.27)*	*1.72 (1.32, 2.23)*	*1.50 (1.10, 2.04)*	*1.59 (1.11, 2.29)*
41–60	0.87 (0.65, 1.17)	1.11 (0.82, 1.52)	1.11 (0.78, 1.57)	*1.77 (1.19, 2.64)*
61–80	Ref	Ref	Ref	Ref
Land cover type				
Urban	*1.45 (1.25, 1.69)*	*1.31 (1.1, 1.56)*	*1.54 (1.27, 1.87)*	1.25 (0.99, 1.57)
Non-urban	Ref	Ref	Ref	Ref
Census community designation				
Urban	*1.31 (1.12, 1.52)*	1.04 (0.88, 1.24)	*1.77 (1.45, 2.16)*	*1.35 (1.07, 1.72)*
Rural	Ref	Ref	Ref	Ref

Adjusted models include age, smoking, BMI, physical activity, stress score, socioeconomic score, and energy intake. Italic type indicates statistically significant associations

Changes in HOMA-IR are estimated for an IQR increase in distance to urban center (32.6 km). We also examined the modification of the association between distance to urban center and HOMA-IR by age, smoking, BMI, physical activity and energy intake in sex-stratified multivariate models (Additional file 2: Figure 1). Results show a significant increase in HOMA-IR the closer participants resided to the urban center of Chennai, with 0.19 mg/dL (95% CI 0.13, 0.25) and 0.16 mg/dL (95% CI 0.09, 0.22) increase in HOMA-IR per IQR increase in distance in females and males, respectively. The estimated change in HOMA-IR corresponding to a distance of 80 km from city center is 23% of the mean for females and males, 0.46 mg/dL and 0.39 mg/dL, respectively. The association was more pronounced in younger females who had a 0.25 mg/dL (95% CI 0.16, 0.35) increase in HOMA-IR for an IQR change in distance compared to 0.15 mg/dL (95% CI 0.03, 0.25) for older females. Closer proximity to the urban center was associated with a higher effect on HOMA-IR among obese males (0.28 mg/dL [95% CI 0.16, 0.43]) compared to non-obese males (0.09 mg/dL [95% CI 0.16, 0.43]). However, effects between obese and non-obese females were similar. In both males and females there was a greater effect of distance on HOMA-IR for participants reporting moderate and low physical activity compared to high physical activity, although there was a high degree of overlap in confidence intervals.

A sensitivity analysis was conducted that replaced BMI with WC in the adjusted logistic regression models (Additional file 1: Table 1). The models adjusting for WC had a significantly lower magnitude of association between an IQR distance from urban center in males (0.09 mg/dL [95% CI 0.16, − 0.013]) than in the models adjusting for BMI, but was not significantly different in females (− 0.16 mg/dL [95% CI − 0.22, − 0.09]).

## Discussion

In a population-based representative sample of adults in India without diabetes we investigated the association between residing in an urban environment and insulin resistance, which is an important underlying metabolic condition predisposing the development of diabetes [32, 33]. After controlling for age, BMI, energy intake, SE score, physical activity, stress and smoking status, there were independent associations between multiple metrics of urban environment and HOMA-IR. Those residing in urban areas as defined by land cover and census category had higher HOMA-IR levels than those in rural or non-urban areas. The largest increase was found for participants living within 20 km of the city center.

In multivariate models there were gender-specific differences of the effect of age and obesity on the association between distance from the urban center and HOMA-IR such that the association was more pronounced in younger females and among obese males. Previous, studies in young populations suggest that girls are intrinsically more insulin resistant [34]. Further, reports show that type 2 diabetes in younger populations show a female preponderance [35–37]. However, at older ages with increases in BMI, there is a greater amounts of visceral and hepatic adipose tissue in males, when compared with females, which contributes to higher insulin resistance in males [38]. These findings are consistent with the greater effect modification of the relationship between distance from urban center and HOMA-IR among younger females and obese males that we found.

A small number of studies have evaluated the impact of urbanization on insulin resistance in varied locations across the globe. A higher prevalence of insulin resistance was identified in Floresian men (a specific ethnic group in Indonesia) that had moved to an urban center (Jakarta) compared to men than remained in the rural area [39]. Similarly, another study revealed statistically significant higher HOMA-IR in Ghanaian adults living in urban areas compared to rural areas [40].

Due to urbanization in India, environmental degradation has been occurring very rapidly resulting in poor water quality, air pollution, noise, dust and heat, as well as problems with disposal of solid and hazardous wastes [9]. Thirteen of the world’s 20 cities with the highest levels of particulate matter less than 2.5 μm in aerodynamic diameter (PM_2.5_) are located within India. Significant sources of air pollution in India include motor vehicles, electricity generation, manufacturing, construction and road dust, which have increased in India’s cities in recent years along with the rapid growth in industry, power and transportation [41]. Air pollution, specifically PM_2.5_ and nitrogen dioxide, have achieved recent attention given associations with diabetes and insulin resistance in multiple studies [10, 20, 42, 43]. Proposed mechanisms for these effects include oxidative stress; endothelial dysfunction; overactivity of the sympathetic nervous system; changes in immune response in visceral adipose tissues; and altered insulin sensitivity and glucose metabolism [42, 43]. Other chemicals such as persistent organic pollutants and endocrine disruptors have also been associated with diabetes [11]. These chemicals may act as antagonists or agonists to endogenous hormones necessary to maintain homeostasis or affect normal functioning of mitochondria [44]. However, it is important to note the spatial variation and temporality between increased pollution and this health effect, because there may be a lag between further degradation of the environment and diagnosis of adverse effects. Understanding the relationship between pollution and insulin resistance will require a more detailed analysis of these temporal trends.

Other contributors to this association are also possible including access to qualified medical care. Past research has found that although there is a greater concentration of medical workers in urban areas a large proportion of those practitioners are also unqualified [45]. Diet is a key factor in insulin resistance and evidence of differences comparing urban and rural populations is mixed. One study reported similar fruit and vegetable intake among both populations [46], another reported high intake of fruits and vegetables, along with higher intake of carbohydrates, meat and dairy for urban populations [47].

One of the strengths of our study is the use of three metrics to test the associations between urban environment and HOMA-IR. Land cover classification allowed us to reduce exposure misclassification by identifying smaller or developing urban enclaves outside of the city center. For example, we could identify a rapidly urbanizing municipality approximately 65 km southwest from the urban center (Fig. 1) that was classified as rural in the India Census data. Female participants residing in this second urban cluster had a mean HOMA-IR of 1.83 mg/dL (SD: 2.17 mg/dL), which was significantly higher than female participants residing in the same distance interval (60–80 km distance group), who had a mean HOMA-IR of 1.57 mg/dL (SD: 0.97 mg/dl). It is possible that the second urban cluster introduced exposure misclassification in the multivariate analysis based on Census classification resulting in a null effect (Table 3). However, examining three metrics reveals an overall commonality of the association between urban residence and higher HOMA-IR.

Our study also has several limitations. Although the land cover data are able to identify rapidly developing urban areas we must compare data for temporally close, but different, years. This will result in some error, which we have sought to address by gathering data on exposure before outcome. Also, the land cover classifications do not differentiate specific land uses within urban areas. There are likely to be differential exposures comparing residential versus industrial land use that may be important to the outcome. Another potential limitation is our geocoding method. Geocoding participant locations can be difficult in rapidly developing regions in India without reliable address network systems and Global Position System (GPS) ascertainment is not viable with large sample populations. Exposure misclassification from positional error could affect our analysis at the edges of our distance interval cut points, as well as with the 500 m × 500 m MODIS land cover grids. Nevertheless, in a subset of participants for whom we compared the geocoded location to the location recorded from a GPS, the mean difference was 0.19 km which is relatively small compared to the 20 km distance categories used in our main analysis. We would anticipate this error to be non-differential with respect to our outcome and therefore would be expected to bias results towards the null.

We found that adjustment for WC instead of BMI resulted in an attenuation of effects among males. This indicates possible residual confounding when using BMI as the measure for adiposity, which may not adequately account for fat distribution. Finally, the study design of our analysis was cross-sectional. We are therefore limited in our ability to evaluate temporality with regard to urban expansion and the effect of urbanization on HOMA-IR. Future work with this cohort may allow us to draw stronger conclusions about which aspects of the urban environment may be most important to the association with HOMA-IR and whether there is a causal association. Future analyses could consider ambient and household air pollution, which are often pervasive, persistent and exist at higher concentrations in urban areas of India [9].

## Conclusion

We have identified independent associations between the urban environment and insulin resistance in a cohort of adults in Southern India. The association was robust using various matrices of urbanization and adjustment for individual predictors. Our results are of public health concern due to the movement of large numbers of people from rural to urban areas in many parts of the world and the already large burden of diabetes. Further research is needed including longitudinal follow-up, to assess the aspects within the urban environment that may be most important to the association with HOMA-IR in this cohort.

## Additional files

**Additional file 1.** Association between distance to urban center and HOMA-IR level comparing the inclusion of either of two measures of adiposity: body mass index (BMI) or waist circumference.

**Additional file 2.** Effect modification of the relationship between distance to urban center and HOMA-IR.

## Abbreviations

BMI: body mass index
CVD: cardiovascular disease
FBS: fasting blood sugar
GPS: Global Position System
HOMA-IR: Homeostasis model assessment of insulin resistance
IQR: interquartile range
OR: odds ratio
PM_2.5_: particulate matter less than 2.5 μm in aerodynamic diameter
PURSE-HIS: Population Study of Urban, Rural, and Semi-urban Regions for the Detection of Endovascular Disease and Prevalence of Risk Factors and Holistic Intervention Study
SE score: socioeconomic score
WC: waist circumference
