# Supplementary material for: Urban environment as an independent predictor of insulin resistance in a South Asian population
Source: Int J Health Geogr. 2019 Feb 12;18:5. doi: 10.1186/s12942-019-0169-9 (PMC6373002; doi:10.1186/s12942-019-0169-9)
Supplement: Supplementary file 1 — Additional file 1. Association between distance to urban center and HOMA-IR level comparing the inclusion of either of two measures of adiposity: body mass index (BMI) or waist circumference. [file 12942_2019_169_MOESM1_ESM.docx]

**Supplemental Table 1:** Association between distance to urban center and HOMA-IR level comparing the inclusion of either of two measures of adiposity: body mass index (BMI) or waist circumference.

| **Covariate included in model** | **Females** | **Males** |
| --- | --- | --- |
|  | β (95%CI) | β (95%CI) |
| BMI | 0.19 (0.13, 0.25) | 0.16 (0.06, 0.25) |
| Waist Circumference | 0.16 (0.09, 0.22) | 0.09 (0.013, 0.16) |
| All models are adjusted for age, smoking, physical activity, stress score, socioeconomic score, and energy intake. | | |
