# Supplementary figures and images for: Urban environment as an independent predictor of insulin resistance in a South Asian population
Source: Int J Health Geogr. 2019 Feb 12;18:5. doi: 10.1186/s12942-019-0169-9 (PMC6373002; doi:10.1186/s12942-019-0169-9)

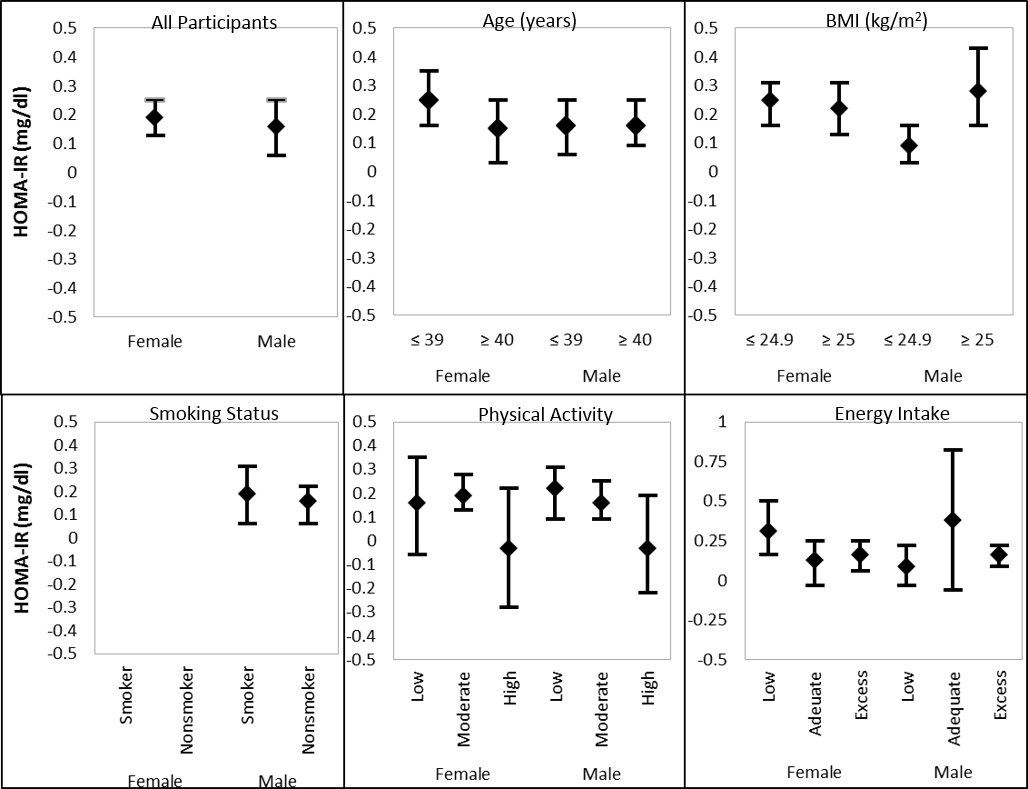

Supplement: Supplementary file 2 — Additional file 2. Effect modification of the relationship between distance to urban center and HOMA-IR. [file 12942_2019_169_MOESM2_ESM.tif]
